# Cadmium exposure and its association with serum uric acid and hyperuricemia

**DOI:** 10.1038/s41598-017-00661-3

**Published:** 2017-04-03

**Authors:** Honglin Sun, Ningjian Wang, Chi Chen, Xiaomin Nie, Bing Han, Qin Li, Chunfang Zhu, Yi Chen, Fangzhen Xia, Yingchao Chen, Hualing Zhai, Boren Jiang, Bin Hu, Yingli Lu

**Affiliations:** 1 Institute and Department of Endocrinology and Metabolism, Shanghai Ninth People’s Hospital, Shanghai JiaoTong University School of Medicine, Shanghai, China; 2 Department of Prosthodontics, Shanghai Ninth People’s Hospital, Shanghai JiaoTong University School of Medicine, Shanghai, China

## Abstract

Few studies have investigated the association between serum uric acid (UA) and cadmium exposure. Our previous study revealed a significantly higher blood cadmium (CdB) level in the Chinese population compared to populations in other countries. To determine whether CdB in Chinese adults is associated with serum UA and hyperuricemia, 2996 participants from the cross-sectional SPECT-China study were recruited. CdB was measured by atomic absorption spectrometry. Hyperuricemia was defined as a serum UA concentration ≥416.4 μmol/L for men and ≥356.9 μmol/L for women. Regression analyses were used to analyze the association of CdB with serum UA and hyperuricemia. We found that the median CdB level was higher in men with hyperuricemia (2.40 μg/L) than in men without hyperuricemia (1.98 μg/L, *P* < 0.05). A positive relationship between serum UA and CdB was found in Chinese men after adjusting for the estimated glomerular filtration rate (eGFR), current smoking status, diabetes, dyslipidemia, hypertension and body mass index and in participants with eGFR > 60 mL/min per 1.73 m^2^. Further, the odds ratio of hyperuricemia increased with increasing CdB quartiles (*P* for trend < 0.05) in men. In conclusion, CdB was positively related to the serum UA level and to hyperuricemia in Chinese men but not in Chinese women.

## Introduction

Serum uric acid (UA) is the final enzymatic product when the body breaks down purine^1^. Increased production or decreased excretion of UA causes hyperuricemia^2^. Previous studies have indicated that hyperuricemia is associated with cardiovascular diseases^3^ and metabolic diseases such as diabetes^4^, hypertension^5^ and dyslipidemia^6^. In past decades, the prevalence of hyperuricemia has increased to 21% and 13% in the US and Chinese general populations, respectively. Although this trend may be related to the increasing prevalence of adiposity and hypertension^1, 4^, environmental factors cannot be ignored.

Cadmium is a toxic metal with negative effects on health^7^. Occupational exposure is mainly from industrial processes. Smoking tobacco and contaminated food such as vegetables and rice are the main sources of general cadmium exposure^7^. Blood cadmium (CdB) levels vary by region, age and ethnicity^7^. Previous studies have confirmed the pathogenic role of cadmium exposure in renal damage^7^, bone destruction^8^ and cancer^9, 10^. Recent research has focused on the role of cadmium as an important environmental endocrine disruptor^11^. Epidemiological studies have linked cadmium exposure to metabolic diseases such as diabetes^12^, obesity^13^ and thyroid disease^14^, although the results have not been consistent^15, 16^. We also found a relationship between cadmium exposure and prediabetes in our previous work^17^. However, the relationship between cadmium exposure and hyperuricemia remains unknown.

Due to its long biological half-life^18^, cadmium mainly accumulates in the kidney and liver of human bodies, which may lead to elevated plasma uric acid levels according to several animal studies^19, 20^. Furthermore, as mentioned above, cadmium exposure is associated with metabolic diseases and thus may prompt the occurrence of hyperuricemia and gout^21, 22^. Epidemiological evidence of a relationship between cadmium exposure and hyperuricemia is scarce. A cross-sectional study from the National Health and Nutrition Examination Survey (NHANES) showed no relationship between them^2^. Nevertheless, our previous study revealed that the CdB level (median of 1.70 μg/L) was much higher than that reported in other countries^17^, and approximately 17% of subjects still had a CdB level higher than 5.0 µg/L. No study has ever explored this association in the Chinese population at the current CdB level, which differs from the level in the US. Hence, using data from a population-based investigation called the Survey on Prevalence in East China for Metabolic Diseases and Risk Factors (SPECT-China) in 2014, we aimed to explore the relationships between the CdB and serum UA levels and hyperuricemia in the general Chinese population.

## Results

### Characteristics of participants by hyperuricemia status

The characteristics of the study population, categorized by sex and hyperuricemia status, are provided in Table 1. Participants with hyperuricemia were more likely to have comorbid conditions such as obesity, hypertension, dyslipidemia and reduced renal function in both genders (*P* < 0.05). The median CdB level was 2.40 (0.68–4.61) μg/L higher in men with hyperuricemia than in men without hyperuricemia (*P* < 0.05), but the CdB levels in women showed no significant difference between individuals with and without hyperuricemia. Additionally, the median blood lead (PbB) levels were comparable between different serum UA levels.

**Table 1:** Characteristics of the participants categorized by hyperuricemia status.

	No hyperuricemia	Hyperuricemia	*P*
***Men***			
*N*	944	291	
Serum UA level, μmol/L	334.0 ± 52.5	473.7 ± 56.3	<0.001
Age, yr	52 ± 14	51 ± 14	0.40
Anthropometric measures			
Height, cm	168.5 ± 7.0	168.8 ± 6.8	0.57
Weight, kg	69.2 ± 10.8	74.4 ± 10.6	<0.001
BMI, kg/m^2^	24.3 ± 3.3	26.1 ± 3.0	<0.001
Waist circumference, cm	82.7 ± 9.5	86.8 ± 7.8	<0.001
Comorbid conditions			
Diabetes, %	14.5	12.4	0.39
Hypertension, %	36.4	47.8	<0.01
Dyslipidemia, %	31.3	54.3	<0.001
CdB level, μg/L	1.98 (0.56–4.19)	2.40 (0.68–4.61)	<0.05
PbB level, μg/L	36.89 (25.15–53.00)	39.00 (25.00–53.68)	0.63
eGFR, mL/min per 1.73 m^2^	84.7 ± 14.0	78.9 ± 16.5	<0.001
Current smoker, %	41.5	46.0	0.18
Rural/urban residence, %	14.2/85.8	14.8/85.2	0.80
***Women***			
*N*	1584	177	
Serum UA level, μmol/L	258.9 ± 48.2	400.3 ± 48.7	<0.001
Age, yr	50 ± 14	59 ± 13	<0.001
Anthropometric measures			
Height, cm	156.9 ± 6.0	155.8 ± 6.5	<0.05
Weight, kg	57.6 ± 8.7	61.7 ± 9.5	<0.001
BMI, kg/m^2^	23.4 ± 3.3	25.4 ± 3.5	<0.001
Waist circumference, cm	74.6 ± 9.6	82.3 ± 9.4	<0.001
Comorbid conditions			
Diabetes, %	7.4	19.8	<0.001
Hypertension, %	27.4	52.4	<0.001
Dyslipidemia, %	19.7	47.5	<0.001
CdB level, μg/L	1.62 (0.50–3.89)	1.71 (0.50–3.66)	0.70
PbB level, μg/L	33.19 (22.00–46.76)	36.00 (22.00–49.60)	0.29
eGFR, mL/min per 1.73 m^2^	87.1 ± 15.4	72.3 ± 17.4	<0.001
Current smoker, %	2.5	6.8	<0.01
Rural/urban residence, %	17.2/82.8	28.8/71.2	<0.001

Data are summarized as the mean ± SD or median (interquartile range) for continuous variables or as a number with a proportion for categorical variables.

Dyslipidemia was defined as total cholesterol ≥6.22 mmol/L, triglycerides ≥2.26 mmol/L, LDL-C ≥4.14 mmol/L or HDL-C <1.04 mmol/L.

Hyperuricemia was defined as a serum urate level ≥416.4 μmol/L in men and ≥356.9 μmol/L in women.

### Association of serum UA level with CdB by linear regression

Linear regression modeling of the data showed that a higher CdB level was associated with a higher serum UA concentration (B = 2.963, p < 0.05) in men after adjusting for PbB (Table 2). This positive correlation remained even after the data were adjusted for estimated glomerular filtration rate (eGFR), current smoking status, diabetes, dyslipidemia, hypertension and body mass index (BMI) (B = 2.718, p < 0.05). After we excluded participants with renal impairment (eGFR ≤ 60 mL/min per 1.73 m^2^) and smokers, the serum UA level remained positively associated with CdB (B = 2.595, p < 0.05 and B = 2.771, p < 0.05, separately). However, no relation between the CdB and serum UA levels was observed in women in either the crude or the fully adjusted model. Furthermore, PbB was analyzed as an independent variable, and we found no correlation between PbB and serum UA levels in either gender.

**Table 2:** Association of CdB level (independent variable) with serum urate level (dependent variable).

Serum urate	CdB level	PbB level
***Men***		
Model unadjusted	2.963 (0.922)*	−0.062 (0.102)
Model adjusted for age, smoking	2.905 (0.922)*	−0.059 (0.102)
Full model, overall**	2.718 (0.862)*	−0.090 (0.096)
Full model, eGFR >60 mL/min per 1.73 m^2^	2.595 (0.852)*	−0.039 (0.095)
Full model, non-smoker	2.771 (1.140)*	0.016 (0.118)
***Women***		
Model unadjusted	−0.272 (0.503)	0.100 (0.072)
Model adjusted for age, smoking	0.439 (0.488)	−0.017 (0.070)
Full model, overall**	0.556 (0.452)	−0.013 (0.066)
Full model, eGFR >60 mL/min per 1.73 m^2^	0.452 (0.451)	0.011 (0.067)
Full model, non-smoker	0.477 (0.472)	−0.015 (0.070)

Data are expressed as unstandardized coefficients (standard errors). Linear regression analyses were used. eGFR, estimated glomerular filtration rate.

*Denotes statistical significance at *P* < 0.05.

**Full model included PbB, eGFR (which incorporates age and serum creatinine level), current smoking status, diabetes, dyslipidemia, hypertension and BMI. All variables were categorical except eGFR and BMI, which were entered as continuous measures.

### Association of CdB quartiles with hyperuricemia by logistic regression analyses

In multivariate-adjusted logistic regression analyses (Table 3), the CdB levels were divided by quartile (Q1: ≤0.60; Q2: 0.61–2.09; Q3: 2.10–4.29; Q4: ≥4.30). The Q1 of CdB was used as the reference. Male participants in the highest quartile of CdB had an OR of 1.50 (95% CI, 1.00 to 2.24) for hyperuricemia after adjusting for age, smoking and PbB (*P* for trend < 0.05). After adjusting for eGFR, current smoking status, PbB, diabetes, dyslipidemia, hypertension and BMI, the ORs of Q3 and Q4 CdB for hyperuricemia were 1.82 (95% CI, 1.18, 2.79) and 1.61 (95% CI, 1.04, 2.49), respectively (*P* for trend < 0.05). Higher ORs for the CdB levels in Q3 (OR = 1.99, 95% CI, 1.26, 3.15) and Q4 (OR = 1.77, 95% CI, 1.11, 2.80) were observed in participants with relatively normal renal function (eGFR > 60 mL/min per 1.73 m^2^, *P* for trend < 0.01). After we excluded smoking participants, a marginal significance for CdB as a risk factor for hyperuricemia remained (*P* for trend = 0.08). In women, the CdB levels were still not related to hyperuricemia (*P* for trend > 0.05).

**Table 3:** Association of blood cadmium level quartiles with hyperuricemia.

Variable	CdB level				
	Q1	Q2	Q3	Q4	*P* for trend
***Men***					
CdB range, μg/L	≤0.60	0.61–2.09	2.10–4.29	≥4.30	
Hyperuricemia					
Model adjusted for age, smoking, PbB	Ref.	1.22 (0.82, 1.80)	1.43 (0.96, 2.12)	1.50 (1.00, 2.24)	<0.05
Full model, overall*	Ref.	1.22 (0.79, 1.87)	1.82 (1.18, 2.79)	1.61 (1.04, 2.49)	<0.05
Full model, eGFR >60 mL/min per 1.73 m^2^	Ref.	1.29 (0.82, 2.03)	1.99 (1.26, 3.15)	1.77 (1.11, 2.80)	<0.01
Full model, non-smoker	Ref.	1.03 (0.58, 1.81)	1.65 (0.94, 2.92)	1.50 (0.85, 2.64)	0.08
***Women***					
CdB range, μg/L	≤0.50	0.51–1.65	1.66–3.88	≥3.89	
Hyperuricemia					
Model adjusted for age, smoking, PbB	Ref.	0.95 (0.60, 1.51)	1.24 (0.79, 1.94)	1.13 (0.70, 1.83)	0.424
Full model, overall*	Ref.	1.02 (0.61, 1.70)	1.36 (0.83, 2.22)	1.25 (0.74, 2.11)	0.265
Full model, eGFR >60 mL/min per 1.73 m^2^	Ref.	0.98 (0.57, 1.67)	1.11 (0.64, 1.94)	1.11 (0.63, 1.94)	0.642
Full model, non-smoker	Ref.	1.08 (0.64, 1.81)	1.33 (0.81, 2.20)	1.11 (0.65, 1.90)	0.53

Data are expressed as odds ratios (confidence interval). Logistic regression analyses were used.

eGFR, estimated glomerular filtration rate.

*Full model included eGFR (which incorporates age and serum creatinine level), current smoking status, PbB, diabetes, dyslipidemia, hypertension and BMI. All variables were categorical except eGFR, PbB and BMI, which were entered as continuous measures.

## Discussion

We explored the association between CdB and UA in Chinese adults. Our study revealed that CdB was positively associated with serum UA levels and hyperuricemia in Chinese men but not in women. This association was independent of PbB, eGFR, current smoking status, diabetes, dyslipidemia, hypertension and BMI. Furthermore, in male participants with relatively normal renal function (eGFR > 60 mL/min per 1.73 m^2^), a positive relationship between CdB and hyperuricemia remained.

Cadmium exposure has been linked to numerous human health problems^11^. Cadmium has been found to target the kidneys and induce proximal tubular reabsorptive dysfunction^7^. Prolonged exposure to high cadmium levels has given rise to osteomalacia as well as osteoporosis^7^. In particular, various studies have demonstrated the possible role of cadmium as an endocrine disruptor^11, 14, 15^. Cadmium can accumulate in the thyroid gland. Colloid cystic goiter, diffuse parafollicular cells, nodular hyperplasia and hypertrophy are often found in chronic cadmium toxicity^14^. Both animal studies and epidemiology studies have revealed that cadmium alters various blood sex hormone levels, such as luteinizing hormone, progesterone and testosterone^11, 23^. Moreover, cadmium can exert an estrogenic effect both *in vivo* and *in vitro* ^24^. Cadmium has been found to accumulate in the pancreas and exhibit detrimental effects on β cell function^25^. Both NHANES and our previous study showed that CdB level was associated with prediabetes^12, 17^.

The CdB levels of our participants were higher than in those in developed countries^18, 26–28^, which may be attributed to the economic boom and industrialization^29^. Industrial uses have led to the widespread dispersion of Cd at trace levels into the air, water, and soil and thus into foods^18^. Atmospheric Cd emissions from non-ferrous metal smelting and coal combustion in China increased by approximately 4.6 fold from 1990 to 2010^29, 30^. Another explanation is dietary habits. As in other Asian countries such as Bangladeshi and Korea^26, 31^, our staple foods are rice and vegetables, which are more likely to be contaminated by cadmium pollution^7^. Furthermore, participants living in areas with low economic status had higher CdB than participants in high-economic-status areas^17^. Industrial factories prefer to build sites in low-economic-status areas because of the low prices of land and labor. Poor infrastructure construction and environmental supervision systems combined with a lack of water-quality monitoring together led to water cadmium contamination^17^.

Little is known about the association of cadmium exposure with UA. The NHANES data for 2005–2008 revealed no relationship between cadmium and gout in the USA^2^. However, the CdB levels in Americans were much lower than those in Chinese adults^17^. Previous animal studies have established models of renal toxicity with decreased eGFR upon cadmium administration, which resulted in elevated serum UA levels^32, 33^. Nevertheless, in our research, CdB was positively correlated with hyperuricemia after adjustment for eGFR and in participants with an eGFR > 60 mL/min per 1.73 m^2^, suggesting other mechanisms beyond a decreased eGFR.

UA is primarily produced in the liver by xanthine oxidoreductase^34^ and then undergoes glomerular filtration, tubular reabsorption and excretion by the kidneys^35^. The excretion of UA consists of a basolateral uptake step mediated by an organic anion transporter^36^, followed by an efflux step mediated by multidrug resistance protein 4 and the urate transporter^37^. Cadmium-related renal damage begins with proximal renal tubular injury^38^ before glomerular injury. Tubular organic anion uptake transporters may be a target for cadmium^19, 33^ because sub-chronic cadmium intoxication results in a loss of basolateral invaginations and the down-regulation of organic anion transporters and organic cation transporters, which may lead to decreased urate secretion from the tubular cells. Cadmium toxicity may lead to impaired p-aminohippurate excretion due to a loss of organic anion carriers in the proximal tubular basolateral membranes^20^. Therefore, we hypothesized that the early renal damage by cadmium exposure might lead to a defect in urate excretion and give rise to hyperuricemia.

Oxidative stress is among the important mechanisms of cadmium toxicity, and the liver is a critical target organ^39^. There is an increased conversion of xanthine oxidoreductase from xanthene dehydrogenase to xanthine oxidase in the cadmium-treated liver^40^. The transition from purine to UA, mediated by xanthine oxidase, leads to the production of reactive oxygen species, which may be accompanied by increased UA production. Furthermore, previous studies have suggested that serum UA is an antioxidant^41^. Hence, elevated serum UA may be a protective mechanism against oxidative stress from cadmium exposure.

The gender-specific association between CdB and UA level is inconclusive. Sex hormones may be involved. CdB was found in a previous study to negatively correlate with total testosterone and sex hormone binding globulin in Chinese men^23^. Conversely, the data from NHANES 2011–2012 show significantly positive associations between CdB and serum testosterone in men^42^. Moreover, estrogen-induced increases in the fractional excretion of UA were associated with lower levels of UA in male-to-female transsexuals^43^. A previous study showed that women and men differed in their pathogenic factors and treatment monitoring because female patients had greater co-morbidities and received the appropriate treatment more often^44^. Knowledge on this gender-specific association is thus rather limited.

Cadmium and lead (Pb) are two toxic metals that are widely distributed in the environment. They share similar population exposure routes^45, 46^. Concurrent exposure to both metals is very common^46, 47^. Epidemiological evidence has shown that CdB is positively related to PbB^17, 48^ and that the two metals have interactive effects in certain diseases^45, 49^. Lead toxicity (>80 μg/dL) is associated with hyperuricemia and gout^47, 50, 51^. Moreover, there is still a link between relatively lower PbB and hyperuricemia^2, 52^. Thus, we regarded PbB as an important confounding factor. Moreover, we evaluated the relationship between PbB and UA levels in our participants, but there was no significant relationship in either men or women.

This study is the first exploration of the relationship between CdB and hyperuricemia in different genders in the Chinese population. Homogeneity and strict quality control were guaranteed because the same trained staff was used. Furthermore, we considered PbB to be a confounding factor when exploring the association between CdB and UA levels.

There are some limitations of this study. First, using a cystatin C-based formula to adjust for the GFR estimates is required in healthy populations with normal renal function, which was not available to us, but the CKD-EPI equation applied in our study was confirmed to be more accurate than the Modification of Diet in Renal Disease Study equation, particularly for censoring numerical estimates greater than 60 mL/min per 1.73 m^2^ ^53^. Second, we used the blood cadmium levels rather than urinary cadmium. Urinary cadmium reflects lifetime cadmium exposure, but for relatively low cadmium exposure levels, blood cadmium levels may be more appropriate^38^. It would have been ideal if we could detect both. Third, this study did not include information on food intake. A high serum UA level is usually associated with an intake of large amounts of food that is high in purines^2^. It is reasonable that the CdB levels are parallel with the serum UA levels in participants with large daily food intakes. Furthermore, we could not determine the causal relationship between CdB and hyperuricemia in this cross-sectional study.

In conclusion, CdB was positively associated with serum UA levels and hyperuricemia in Chinese men but not in women. This study indicated that cadmium exposure may confer a risk for hyperuricemia, which was not attributed solely to cadmium toxicity-induced renal dysfunction. However, in cases of relatively normal renal function, the CdB level was still positively related to serum UA. Further study is needed to demonstrate causality and elucidate the underlying mechanisms. In addition, efforts to reduce cadmium exposure in adults are warranted.

## Methods

### Study population

Our data (n = 6899) were from the SPECT-China study^54, 55^. The sampling method was described in detail in our previous study^23^. A total of 2996 subjects were enrolled in our final study after excluding participants with missing values for UA (n = 3429) and CdB (n = 474). Before the data collection, written informed consent was provided by all participants. All procedures followed were in accordance with the ethical standards of the responsible committee on human experimentation (institutional and national) and with the Helsinki Declaration of 1975, as revised in 2008. The study protocol was approved by the Ethics Committee of Shanghai Ninth People’s Hospital, Shanghai Jiao Tong University School of Medicine.

### Measurements

The questionnaires about demographic characteristics, medical history and lifestyle risk factors and anthropometric data were constructed by the same trained staff as previously described^54, 55^. Body weight, height and the calculation of BMI were calculated consistently with the previous study^23^. Waist circumference and blood pressure were measured by strict adherence to the standard procedure^21^. Current smoking was defined as having smoked at least 100 cigarettes in one’s lifetime and currently smoking cigarettes^17^.

Venous blood samples were drawn, processed and shipped as previously described^54, 55^. Serum UA levels were measured using the uricase method with a Beckman Coulter AU 680 (Germany). The coefficient of variation was between 1.2% and 2.7%. Serum creatinine (Scr) was measured using a kinetic-rate Jaffe method with a Beckman Coulter AU 680 (Germany), and we converted the Scr levels to the estimated glomerular filtration rate (eGFR) according to the Chronic Kidney Disease Epidemiology Collaboration (CKD-EPI) equation: (1) for a woman with Scr ≤0.7 mg/dL, eGFR = 144 × (Scr/0.7)^−0.329^ × (0.993)^age^; (2) for a woman with Scr >0.7 mg/dL, eGFR = 144 × (Scr/0.7)^−1.209^ × (0.993)^age^; (3) for a man with Scr ≤0.9 mg/dL, eGFR = 141 × (Scr/0.9)^−0.411^ × (0.993)^age^; and (4) for a man with Scr >0.9 mg/dL, eGFR = 141 × (Scr/0.9)^−1.209^ × (0.993)^age^. The value of eGFR is reported in units of mL/min per 1.73 m^2^ of body surface area^17^.

Cadmium and lead levels in blood samples were tested using graphite furnace atomic absorption spectrometry^17^. Standard curves were established, and quality control materials were tested before the samples were measured. Two quality control personnel participated in the process control. Outliers were detected by duplicate runs. The detection limits for blood cadmium and lead were 0.01 µg/L and 0.1 µg/L, respectively. The inter-assay coefficient of variation for cadmium was 10%.

Fasting plasma glucose (FPG), total cholesterol (TC), triglycerides (TG), high-density lipoprotein (HDL), low-density lipoprotein (LDL), insulin and glycated hemoglobin (HbA1c) were assessed with the methods used previously^17^.

### Definition of variables

Hyperuricemia was defined as a serum UA concentration ≥416.4 μmol/L and ≥356.9 μmol/L for men and women, respectively^2^. The definitions of overweight, obese, diabetic and hypertensive in this study have been previously described^21^. Dyslipidemia was defined as described previously^56^.

### Statistical analyses

The IBM SPSS Statistics software, version 22 (IBM Corporation, Armonk, NY, USA), was used for data analysis. Analyses were performed separately for men and women due to major gender differences in serum UA concentrations. A *P* value < 0.05 for a two-tailed test indicated a significant difference. The specific statistical methods for continuous variables and categorical variables were described in detail in a previous study^17^.

The association of CdB (an independent variable) with serum UA levels (a dependent variable) was assessed by linear regression analysis. The results were expressed as unstandardized coefficients (*B*) and standard errors. The full model included PbB, eGFR (which incorporates age and serum creatinine level), current smoking status, diabetes, dyslipidemia, hypertension and BMI.

To consider the association of CdB with hyperuricemia, logistic regression analyses were used. CdB was divided into quartiles, with the first quartile representing the lowest levels and the fourth quartile the highest. The full model included eGFR (which incorporates age and serum creatinine levels), current smoking status, PbB, diabetes, dyslipidemia, hypertension and BMI. PbB, eGFR, and BMI were entered as continuous measures. Data were expressed as odds ratios (ORs) (95% confidence interval (CI).

### Subgroup analyses

Because hyperuricemia is known to be associated with kidney dysfunction and the kidneys are the most important target organs for cadmium exposure, we performed subgroup analyses that excluded participants with an eGFR of 60 mL/min per 1.73 m^2^ or less^2^. Moreover, smokers are at high risk of cadmium exposure^17^, and previous studies have indicated an association between smoking and increased purine catabolism^57^. Thus, we performed another subgroup analysis excluding current smokers. The regressions were performed by the same strategy as in the above analyses.

## References

[1] Liu R (2015). Prevalence of Hyperuricemia and Gout in Mainland China from 2000 to 2014: A Systematic Review and Meta-Analysis. BioMed research international.

[2] Krishnan E, Lingala B, Bhalla V (2012). Low-level lead exposure and the prevalence of gout: an observational study. Annals of internal medicine.

[3] Bos MJ, Koudstaal PJ, Hofman A, Witteman JC, Breteler MM (2006). Uric acid is a risk factor for myocardial infarction and stroke: the Rotterdam study. Stroke; a journal of cerebral circulation.

[4] Zhu Y, Pandya BJ, Choi HK (2011). Prevalence of gout and hyperuricemia in the US general population: the National Health and Nutrition Examination Survey 2007–2008. Arthritis and rheumatism.

[5] Xu W (2016). Hyperuricemia induces hypertension through activation of renal epithelial sodium channel (ENaC). Metabolism: clinical and experimental.

[6] Peng TC (2015). Relationship between hyperuricemia and lipid profiles in US adults. BioMed research international.

[7] Jarup L, Akesson A (2009). Current status of cadmium as an environmental health problem. Toxicology and applied pharmacology.

[8] Musacchio, E. *et al*. Hyperuricemia, Cardiovascular Profile, and Comorbidity in Older Men and Women: The Pro.V.A. Study. *Rejuvenation research*, doi:10.1089/rej.2016.1834 (2016). 10.1089/rej.2016.1834 27241310

[9] Nawrot T (2006). Environmental exposure to cadmium and risk of cancer: a prospective population-based study. The Lancet. Oncology.

[10] Ali I (2012). Cadmium-induced effects on cellular signaling pathways in the liver of transgenic estrogen reporter mice. Toxicological sciences: an official journal of the Society of Toxicology.

[11] Iavicoli I, Fontana L, Bergamaschi A (2009). The effects of metals as endocrine disruptors. Journal of toxicology and environmental health. Part B, Critical reviews.

[12] Wallia A, Allen NB, Badon S, El Muayed M (2014). Association between urinary cadmium levels and prediabetes in the NHANES 2005–2010 population. International journal of hygiene and environmental health.

[13] Padilla MA, Elobeid M, Ruden DM, Allison DB (2010). An examination of the association of selected toxic metals with total and central obesity indices: NHANES 99-02. International journal of environmental research and public health.

[14] Jancic SA, Stosic BZ (2014). Cadmium effects on the thyroid gland. Vitamins and hormones.

[15] Borne Y (2014). Cadmium exposure and incidence of diabetes mellitus–results from the Malmo Diet and Cancer study. PloS one.

[16] Lee BK, Kim Y (2016). Association of Blood Cadmium Level with Metabolic Syndrome After Adjustment for Confounding by Serum Ferritin and Other Factors: 2008-2012 Korean National Health and Nutrition Examination Survey. Biological trace element research.

[17] Nie, X. *et al*. Blood cadmium in Chinese adults and its relationships with diabetes and obesity. *Environmental science and pollution research international*, doi:10.1007/s11356-016-7078-2 (2016). 10.1007/s11356-016-7078-2 27312901

[18] Garner R, Levallois P (2016). Cadmium levels and sources of exposure among Canadian adults. Health reports.

[19] Ljubojevic M, Breljak D, Herak-Kramberger CM, Anzai N, Sabolic I (2016). Expression of basolateral organic anion and cation transporters in experimental cadmium nephrotoxicity in rat kidney. Archives of toxicology.

[20] Kim YK, Choi JK, Kim JS, Park YS (1988). Changes in renal function in cadmium-intoxicated rats. Pharmacology & toxicology.

[21] Xu Y (2013). Prevalence and control of diabetes in Chinese adults. Jama.

[22] Wang J (2014). Hyperuricemia and risk of incident hypertension: a systematic review and meta-analysis of observational studies. PloS one.

[23] Chen C (2016). Blood Cadmium Level Associates with Lower Testosterone and Sex Hormone-Binding Globulin in Chinese men: from SPECT-China Study, 2014. Biological trace element research.

[24] Garcia-Morales P (1994). Effect of cadmium on estrogen receptor levels and estrogen-induced responses in human breast cancer cells. The Journal of biological chemistry.

[25] El Muayed M (2012). Accumulation of cadmium in insulin-producing beta cells. Islets.

[26] He P (2013). Exposure assessment of dietary cadmium: findings from Shanghainese over 40 years, China. BMC public health.

[27] Heitland P, Koster HD (2006). Biomonitoring of 37 trace elements in blood samples from inhabitants of northern Germany by ICP-MS. Journal of trace elements in medicine and biology: organ of the Society for Minerals and Trace Elements (GMS).

[28] Nisse, C. *et al*. Blood and urinary levels of metals and metalloids in the general adult population of Northern France: The IMEPOGE study, 2008–2010. *International journal of hygiene and environmental health*, doi:10.1016/j.ijheh.2016.09.020 (2016). 10.1016/j.ijheh.2016.09.020 27931767

[29] Cheng K (2015). Atmospheric emission characteristics and control policies of five precedent-controlled toxic heavy metals from anthropogenic sources in China. Environmental science & technology.

[30] Shao X, Cheng H, Li Q, Lin C (2013). Anthropogenic atmospheric emissions of cadmium in China. Atmospheric Environment.

[31] Kim M, Wolt JD (2011). Probabilistic risk assessment of dietary cadmium in the South Korean population. Food additives & contaminants. Part A, Chemistry, analysis, control, exposure & risk assessment.

[32] Dkhil MA (2014). The potential protective role of Physalis peruviana L. fruit in cadmium-induced hepatotoxicity and nephrotoxicity. Food and chemical toxicology: an international journal published for the British Industrial Biological Research Association.

[33] Wang J (2012). Quercetin Protects against Cadmium-Induced Renal Uric Acid Transport System Alteration and Lipid Metabolism Disorder in Rats. Evidence-based complementary and alternative medicine: eCAM.

[34] Ohtsubo T (2009). Xanthine oxidoreductase depletion induces renal interstitial fibrosis through aberrant lipid and purine accumulation in renal tubules. Hypertension.

[35] Van Aubel RA, Smeets PH, van den Heuvel JJ, Russel FG (2005). Human organic anion transporter MRP4 (ABCC4) is an efflux pump for the purine end metabolite urate with multiple allosteric substrate binding sites. *American journal of physiology*. Renal physiology.

[36] Burckhardt G (2012). Drug transport by Organic Anion Transporters (OATs). Pharmacology & therapeutics.

[37] Hu QH, Wang C, Li JM, Zhang DM, Kong LD (2009). Allopurinol, rutin, and quercetin attenuate hyperuricemia and renal dysfunction in rats induced by fructose intake: renal organic ion transporter involvement. American journal of physiology. Renal physiology.

[38] Akesson A (2014). Non-renal effects and the risk assessment of environmental cadmium exposure. Environmental health perspectives.

[39] Matovic V, Buha A, Ethukic-Cosic D, Bulat Z (2015). Insight into the oxidative stress induced by lead and/or cadmium in blood, liver and kidneys. Food and chemical toxicology: an international journal published for the British Industrial Biological Research Association.

[40] Esteves AC, Felcman J (2000). Study of the effect of the administration of Cd(II), cysteine, methionine, and Cd(II) together with cysteine or methionine on the conversion of xanthine dehydrogenase into xanthine oxidase. Biological trace element research.

[41] Alvarez-Lario B, Macarron-Vicente J (2011). Is there anything good in uric acid?. QJM: monthly journal of the Association of Physicians.

[42] Lewis RC, Meeker JD (2015). Biomarkers of exposure to molybdenum and other metals in relation to testosterone among men from the United States National Health and Nutrition Examination Survey 2011–2012. Fertility and sterility.

[43] Yahyaoui R (2008). Effect of long-term administration of cross-sex hormone therapy on serum and urinary uric acid in transsexual persons. The Journal of clinical endocrinology and metabolism.

[44] Harrold LR (2006). Sex differences in gout epidemiology: evaluation and treatment. Annals of the rheumatic diseases.

[45] Hambach R (2013). Co-exposure to lead increases the renal response to low levels of cadmium in metallurgy workers. Toxicology letters.

[46] Wang G, Fowler BA (2008). Roles of biomarkers in evaluating interactions among mixtures of lead, cadmium and arsenic. Toxicology and applied pharmacology.

[47] Ekong EB, Jaar BG, Weaver VM (2006). Lead-related nephrotoxicity: a review of the epidemiologic evidence. Kidney international.

[48] Chen X (2014). Effects of lead and cadmium co-exposure on bone mineral density in a Chinese population. Bone.

[49] Johri N, Jacquillet G, Unwin R (2010). Heavy metal poisoning: the effects of cadmium on the kidney. Biometals: an international journal on the role of metal ions in biology, biochemistry, and medicine.

[50] Dalvi SR, Pillinger MH (2013). Saturnine gout, redux: a review. The American journal of medicine.

[51] Campbell BC, Moore MR, Goldberg A, Hernandez LA, Dick WC (1978). Subclinical lead exposure: a possible cause of gout. British medical journal.

[52] Shadick NA (2000). Effect of low level lead exposure on hyperuricemia and gout among middle aged and elderly men: the normative aging study. The Journal of rheumatology.

[53] Stevens LA (2010). Comparative performance of the CKD Epidemiology Collaboration (CKD-EPI) and the Modification of Diet in Renal Disease (MDRD) Study equations for estimating GFR levels above 60 mL/min/1.73 m^2^. American journal of kidney diseases: the official journal of the National Kidney Foundation.

[54] Wang N (2015). Is Exposure to Famine in Childhood and Economic Development in Adulthood Associated with Diabetes?. The Journal of clinical endocrinology and metabolism.

[55] Wang N (2016). Exposure to Famine in Early Life and Nonalcoholic Fatty Liver Disease in Adulthood. The Journal of clinical endocrinology and metabolism.

[56] Lu J (2014). The relationship between insulin-sensitive obesity and cardiovascular diseases in a Chinese population: results of the REACTION study. International journal of cardiology.

[57] Lain KY, Markovic N, Ness RB, Roberts JM (2005). Effect of smoking on uric acid and other metabolic markers throughout normal pregnancy. The Journal of clinical endocrinology and metabolism.

